# Understanding and managing variation: three different perspectives

**DOI:** 10.1186/1748-5908-8-S1-S1

**Published:** 2013-04-19

**Authors:** Michael E Bowen, Duncan Neuhauser

**Affiliations:** 1 VA National Quality Scholars Fellowship, Tennessee Valley Healthcare System, Nashville, Tennessee, 37212, USA; 2 Division of General Internal Medicine, Department of Medicine, University of Texas Southwestern Medical Center, Dallas, Texas, 75390, USA; 3 Division of Outcomes and Health Services Research, Department of Clinical Sciences, University of Texas Southwestern Medical Center, Dallas, Texas, 75390, USA; 4 Department of Epidemiology and Biostatistics, Case Western Reserve University, Cleveland, Ohio, 44106, USA

## Presentation

Managing variation is essential to quality improvement. Quality improvement is primarily concerned with two types of variation – common-cause variation and special-cause variation. Common-cause variation is random variation present in stable healthcare processes. Special-cause variation is an unpredictable deviation resulting from a cause that is not an intrinsic part of a process. By careful and systematic measurement, it is easier to detect changes that are not random variation.

The approach to managing variation depends on the priorities and perspectives of the improvement leader and the intended generalizability of the results of the improvement effort. Clinical researchers, healthcare managers, and individual patients each have different goals, time horizons, and methodological approaches to managing variation; however, in all cases, the research question should drive study design, data collection, and evaluation. To advance the field of quality improvement, greater understanding of these perspectives and methodologies is needed [1].

### Clinical researcher perspective

The primary goal of traditional randomized controlled trials (RCTs) (ie a comparison of treatment A versus placebo) is to determine treatment or intervention efficacy in a specified population when all else is equal. In this approach, researchers seek to maximize internal validity. Through randomization, researchers seek to balance variation in baseline factors by randomizing patients, clinicians, or organizations to experimental and control groups. Researchers may also increase understanding of variation within a specific study using approaches such as stratification to examine for effect modification. Although the generalizability of outcomes in all research designs is limited by the study population and setting, this can be particularly challenging in traditional RCTs. When inclusion criteria are strict, study populations are not representative of “real world” patients, and the applicability of study findings to clinical practice may be unclear. Traditional RCTs are limited in their ability to evaluate complex processes that are purposefully and continually changing over time because they evaluate interventions in rigorously controlled conditions over fixed time frames [2]. However, using alternative designs such as hybrid, effectiveness studies discussed in these proceedings or pragmatic RCTs, researchers can rigorously answer a broader range of research questions [3].

### Healthcare manager perspective

Healthcare managers seek to understand and reduce variation in patient populations by monitoring process and outcome measures. They utilize real-time data to learn from and manage variation over time. By comparing past, present, and desired performance, they seek to reduce undesired variation and reinforce desired variation. Additionally, managers often implement best practices and benchmark performance against them. In this process, efficient, time-sensitive evaluations are important. Run charts and Statistical Process Control (SPC) methods leverage the power of repeated measures over time to detect small changes in process stability and increase the statistical power and rapidity with which effects can be detected [1].

### Patient perspective

While the clinical researcher and healthcare manager are interested in understanding and managing variation at a population level, the individual patient wants to know if a particular treatment will allow one to achieve health outcomes similar to those observed in study populations. Although the findings of RCTs help form the foundation of evidence-based practice and managers utilize these findings in population management, they provide less guidance about the likelihood of an individual patient achieving the average benefits observed across a population of patients. Even when RCT findings are statistically significant, many trial participants receive no benefit. In order to understand if group RCT results can be achieved with individual patients, a different methodological approach is needed. “N-of-1 trials” and the longitudinal factorial design of experiments allow patients and providers to systematically evaluate the independent and combined effects of multiple disease management variables on individual health outcomes [4]. This offers patients and providers the opportunity to collect, analyze, and understand data in real time to improve individual patient outcomes.

## Commentary

Advancing the field of improvement science and increasing our ability to understand and manage variation requires an appreciation of the complementary perspectives held and methodologies utilized by clinical researchers, healthcare managers, and patients. To accomplish this, clinical researchers, healthcare managers, and individual patients each face key challenges.

## Recommendations

Clinical researchers are challenged to design studies that yield generalizable outcomes across studies and over time. One potential approach is to anchor research questions in theoretical frameworks to better understand the research problem and relationships among key variables. Additionally, researchers should expand methodological and analytical approaches to leverage the statistical power of multiple observations collected over time. SPC is one such approach. Incorporation of qualitative research and mixed methods can also increase our ability to understand context and the key determinants of variation.

Healthcare managers are challenged to identify best practices and benchmark their processes against them. However, the details of best practices and implementation strategies are rarely described in sufficient detail to allow identification of the key drivers of process improvement and adaption of best practices to local context. By advocating for transparency in process improvement and urging publication of improvement and implementation efforts, healthcare managers can enhance the spread of best practices, facilitate improved benchmarking, and drive continuous healthcare improvement.

Individual patients and providers are challenged to develop the skills needed to understand and manage individual processes and outcomes. As an example, patients with hypertension are often advised to take and titrate medications, modify dietary intake, and increase activity levels in a non-systematic manner. The longitudinal factorial design offers an opportunity to rigorously evaluate the impact of these recommendations, both in isolation and in combination, on disease outcomes [1]. Patients can utilize paper, smart phone applications, or even electronic health record portals to sequentially record their blood pressures. Patients and providers can then apply simple SPC rules to better understand variation in blood pressure readings and manage their disease [5].

As clinical researchers, healthcare managers, and individual patients strive to improve healthcare processes and outcomes, each stakeholder brings a different perspective and set of methodological tools to the improvement team. These perspectives and methods are often complementary such that it is not which methodological approach is “best” but rather which approach is best suited to answer the specific research question. By combining these perspectives and developing partnerships with organizational managers, improvement leaders can demonstrate process improvement to key decision makers in the healthcare organization. It is through such partnerships that the future of quality improvement research is likely to find financial support and ultimate sustainability.
